# A Durable Relationship: Respiratory Syncytial Virus Bronchiolitis and Asthma past Their Golden Anniversary

**DOI:** 10.3390/vaccines8020201

**Published:** 2020-04-26

**Authors:** Ignacio Esteban, Renato T. Stein, Fernando P. Polack

**Affiliations:** 1 Fundación INFANT, Buenos Aires 1406, Argentina; 2 Hospital de Pediatría Prof. Dr. Juan P. Garrahan, Buenos Aires 1245, Argentina; 3 Centro INFANT at Pontificia Universidade Catolica de Rio Grande do Sul, Porto Alegre 90610-000, Brasil; 4 Department of Pediatrics, Vanderbilt University, Nashville 37240, Tennessee

**Keywords:** respiratory syncytial virus, bronchiolitis, asthma, wheezing

## Abstract

Numerous preventive strategies against respiratory syncytial virus (RSV) are undergoing late stage evaluation in humans and, in addition to their intended benefit for acute illness, may impact long term consequences of infection in infants. Severe RSV infection has been repeatedly associated in the literature with long term complications, including impaired lung function, recurrent wheezing, and asthma. However, whether RSV lower respiratory tract infection (LRTI) causally affects the odds for developing wheezing and/or asthma during childhood requires further study, and the biological mechanisms underlying this hypothetical progression from viral illness to chronic lung disease are poorly characterized. In this review, we summarize the literature exploring the association between RSV LRTI in infancy and subsequent recurrent wheezing and pediatric asthma.

## 1. Introduction

Respiratory syncytial virus (RSV) is the leading cause of lower respiratory tract infection (LRTI) in infants and young children, causing an estimated 33 million LRTIs, 3.2 to 3.4 million hospitalizations, and 66,000 to 199,000 deaths in children under five years of age, every year, worldwide [1,2]. By the age of two, almost every child has been infected with the virus [3,4]. Numerous preventive strategies against the virus are undergoing late stage evaluation in humans and, in addition to their intended benefit for acute illness, their impact on long term consequences of RSV disease will also be important.

Severe RSV infection has been repeatedly associated in the literature with long term complications, including impaired lung function, recurrent wheezing, and asthma [5,6]. The first references proposing a link between bronchiolitis and recurrent wheezing/asthma date from 1959 [7]. In the last 60 years, numerous studies explored this association, more recently diving into potential causal pathways through probe studies using preventive agents against the virus [8,9].

Wheezing is a non-specific sign at physical examination, caused by the passage of air through narrowed central airways [10]. It is a manifestation of inflammation in the airway mucosa and in the interstitial tissue, associated or not with bronchoconstriction. Risk factors for recurrent wheezing include smoking exposure, parental asthma, daycare attendance, premature birth, lack of breastfeeding, congenital heart disease, and vulnerable socioeconomic status with precarious homes, lack of sewage, or deficient access to clean water [11,12]. Given its high prevalence as a clinical manifestation in different ailments, authors sometimes consider wheezing an independent entity itself, describing it as asthmatic bronchitis, bronchiolitis, or virus-induced wheezing. Repetitive episodes are described as recurrent wheezing, and even as “asthma until proven otherwise” [11].

Infants with severe RSV LRTI early in life are more susceptible to recurrent episodes of wheezing than those who never experienced a severe early episode of lung disease [13,14,15]. In fact, association between severe RSV LRTI and pediatric asthma was repeatedly reported [13,16,17,18]. However, whether RSV LRTI causally affects the odds for developing asthma during childhood remains undetermined, and the biological mechanisms underlying this hypothetical progression from viral illness to chronic lung disease are poorly characterized [19].

Hypotheses based on the timing of RSV infection have been advanced to explain the relationship between the acute viral event and recurrent wheezing/asthma. For instance, severe RSV LRTI typically affects infants and children under the age of two years, when the immune system is still immature and modulation of innate and adaptive responses may be affected by infectious agents [20,21]. In addition, RSV infection and injury when remodeling of the airways and pulmonary parenchyma is at a maximum rate has also been postulated to explain the postulated causal link to asthma [21]. However, most children with RSV LRTI early in life do not progress to develop atopic asthma. Moreover, only 30 to 50% of children diagnosed with asthma have previously experienced a viral LRTI requiring a healthcare visit [19,22]. These observations suggest that for a causal link between both entities to exist, it requires a combination of genetic, environmental, and/or other factors besides the viral infection. Perhaps, instead, RSV may explain one of several wheezing endotypes (wheezing phenotypes with different specific pathogeneses) that coalesce under the “umbrella” we agreed to name “pediatric asthma”. Alternatively, severe RSV LRTI may simply be an early marker for a group of susceptible hosts to be diagnosed as asthmatics later in life [21].

In addition, the recent understanding of asthma not as a disease with a single mechanism of illness but as a syndrome [23,24], reformulates our interpretation of causality previously ascribed to RSV, demanding a recategorization of the specific relationship between “RSV bronchiolitis” and the several different “asthmas” experienced during childhood [25]. In this review, we summarize the literature exploring the association between RSV LRTI in infancy and subsequent recurrent wheezing and pediatric asthma.

## 2. Epidemiological Studies Exploring the Association between RSV LRTI and Asthma

The first manuscript proposing an association between bronchiolitis and asthma dates from 1959, when 100 children were followed for seven years after an episode of bronchiolitis in New York and 32% progressed to develop asthma [7]. Four years later, a second report by Eisen et al. described an incidence of asthma of 25% in a retrospective cohort of 104 patients with acute bronchiolitis; 62% with a family history of allergic manifestations [26]. The first recorded specific description of the association between acute RSV disease and long-term wheezing dates back to 1971, when Rooney et al. followed a cohort of 62 patients after RSV LRTI and 52% experienced recurrent wheezing [16].

The literature is full of reports exploring the association between RSV and recurrent wheezing/asthma. In one study including over 95,000 infants, those children born four months before the winter viral peak had a higher risk of developing asthma at age five, compared to controls [27]. Since RSV is overwhelmingly the most frequent viral pathogen in infants during the winter season, this observation suggests a potential causal link between both entities [27,28]. In the same line of thought, a long-term follow-up study in children with RSV LRTI found 75% of them continued wheezing, 60% with airway obstruction, but interestingly only 16% had a positive response to salbutamol two years after experiencing acute disease [29]. Martinez et al. showed that 60% of children wheezing before the age of three had transient conditions and experienced resolution of symptoms by the age of six. This group of transient wheezers, unlike persistent wheezers with high immunoglobulin E (IgE) and lung function impairment, failed to show associations with allergies, asthma, or diminished lung function [30]. The study was followed by a second prospective report in which RSV LRTI conferred an increased risk of infrequent and frequent wheeze by age six that persisted until age 11, but was no longer detectable in the study population by age 13 [20]. Again, in concordance with other reports, the study found no association between RSV LRTI and atopic status [18,31,32].

In contrast, Sigurs et al. reported in Scandinavia, that when RSV infection was severe enough to lead to hospitalization, it became an independent risk factor for allergic sensitization and asthma inception by age 7.5 years [33]. An Australian prospective cohort of infants with RSV LRTI, followed until the age of six years, observed a cumulative incidence rate of 92% in wheezing symptoms in the five years following enrollment, and a high incidence of familial atopy and bronchial responsiveness to histamine [13]. In line with several of these observations, studies of children with recurrent wheezing after RSV infection detected specific IgE in the respiratory tract [34,35], accompanied by lower lung function [36]. The Childhood Origins of ASThma cohort [37] and the Childhood Asthma Study [38] identified RSV and human rhinovirus lung infections as risk factors for asthma diagnosis, in particular in the presence of allergic sensitization. In light of these results, Sly et al. proposed a “two hit model” of “synergistic interaction” between allergic sensitization and severe viral LRTI in the inception of pediatric asthma [21]. In line with this hypothesis, Martinez et al. identified an interaction between RSV and active smoking for experiencing asthma by age 29 [39].

Finally, using an attractive approach to address the link between RSV LRTI and asthma, a Danish twin cohort study of 8280 pairs reported an association between RSV hospitalization and asthma. However, when modeling the direction of causation, a model postulating asthma as causal for RSV hospitalization fitted better the data, and suggested that RSV hospitalization is not cause of, but an indicator of genetic susceptibility to asthma [18].

## 3. Molecular and Genetic Studies

Numerous studies examined the biological plausibility of RSV infection at an early age as a cause of asthma, from molecular studies of signaling pathways common to both conditions to immunological and genetic studies exploring overlaps in both diseases’ backgrounds. Severity of RSV disease was postulated to affect remodeling of the airways, leading to reactive airways disease and asthma in childhood, and a deficient cellular immune response to RSV was postulated as a contributor by increasing severity of infection [5,40]. In addition, not only the severity of infection, but the number of LRTI episodes elicited by respiratory viruses in the first years of life, were reported to increase the risk for the subsequent development of asthma [37,38,41]. Clinical signs of bronchoconstriction in both entities suggest potential similarities in pathophysiology. However, infants with RSV bronchiolitis often fail to respond to bronchodilators and steroids during acute episodes, in contrast with children with asthma exacerbation [42,43,44,45].

The initial inflammatory response to RSV infection, characterized by an increased production of neutrophils [46,47], reduced natural killer cells [48], and increased concentrations of innate immune proinflammatory Th1 cytokines [47,49] and transient systemic T-cell lymphopenia [50,51] is unusual in pediatric asthma crisis [52]. However, several molecular or immune perturbations were reported, both for RSV severe disease and asthma. These include single nucleotide polymorphisms (SNPs) in genes coding for interleukin responses (IL-4RA, IL-4, IL-13, IL-10, IL-8, and IL-18), TGFβ, TNFα, CX3CR1, surfactant proteins A and D, MS4A2, vitamin D receptor, chemokine receptor, and ligand 5, ADAM33, and in several pattern recognition receptors including TLR 4, TLR 6, TLR7, and TLR10 [5,17,53,54,55,56,57,58]. These findings suggest a shared predisposition for RSV disease severity and later asthma in subjects with specific genetic alterations.

In terms of immune biomarkers, in a prospective cohort of 206 RSV infected infants’ expression of chemokine ligand 5 (CCL 5, also known as RANTES, a chemoattractant for eosinophils, T cells, and basophils) in nasal epithelia during infection was associated with an increased risk for experiencing physician-diagnosed asthma before age seven [54]. In addition, a second cohort of 103 infants with LRTI identified low-IL-10-producing SNPs in association with obstructive parameters using impulse oscillometry, resembling patients with asthma-like symptoms at age six [59]. In the same population, polymorphisms in TLR7 rs179008 are associated with lung function deficiency, and in TLR4 rs4986790 and TLR6 rs5743810, with airways reactivity [56]. In line with the association between TLR4 SNPs and asthma after bronchiolitis, infants with Asp299Gly and/or Thre399Ile in TLR4 experience a skewed Th2 response in the respiratory tract during RSV LRTI [55]. Levels of IL-12p40 and IL-3 during RSV infection in respiratory secretions [60], and persistence of VEGF, IFNγ, GCSF, IL-6, IL-7, IL-10, and IL-13 [61] have also been speculated to serve as potential predictors for recurrent wheezing and/or asthma.

## 4. Pulmonary Function Tests to Investigate the Association between RSV LRTI and Asthma

Lung function tests have served as objective tools in investigation of a heterogeneous disease like asthma, for decades [23,62,63]. In 1977, evaluation of 11-year old children (*n* = 23) enrolled as infants with acute bronchiolitis found 31% with abnormalities in body plethysmograph, spirometry, diffusion capacity for carbon monoxide, and exercise testing [64]. Moreover, 40% had an increased residual volume/total lung capacity, but only 4.5% experienced exercise-induced bronchospasm. These observations suggest that bronchiolitis can lead to prolonged peripheral airway obstruction or loss of elastic recoil plus residual parenchymal injury [64]. A latter study supported these observations, reporting 75% of infants with abnormal lung function a year after having an acute episode of bronchiolitis [32].

Evidence of airway hyperreactivity (a key physiologic feature of asthma), either after exercise, albuterol, methacholine, or histamine challenge, is an important study endpoint to establish a direct link between RSV bronchiolitis and pediatric asthma. However, results of airway hyperreactivity assessments after RSV bronchiolitis remain inconclusive [65,66,67]. In fact, lung function evaluations do suggest that children with RSV bronchiolitis may evolve to experience long-term wheezing more often than asthma. For instance, Soto et al. showed that 30% of infants who experienced severe RSV LRTI had improvement of specific conductance after receiving salbutamol [68]. Yet, in adolescence many of these patients were not diagnosed with clinical asthma despite a high incidence of family history of atopy [13]. In a second study, 57 preschool children with recurrent wheezing after RSV LRTI exhibited declining lung function by adolescence, but no reactive airways after being subjected to a methacholine test [69]. In addition, 1246 children in Arizona with a prior RSV LRTI had diminished forced expiratory volumes, but no significant response to salbutamol [20]. Finally, a longitudinal study following children after RSV bronchiolitis until 18–20 years of age described normal expiratory volumes that were, however, significantly lower than those observed in control subjects [70]. RSV infection was an independent risk factor for lung function abnormalities, even when adjusted for the presence of atopy [70]. In one additional study of 109 children between 17 and 20 years, those who had experienced severe RSV in infancy persisted with lower forced vital capacity (FVC) than those who did not. The incidence of asthma was 43% in those with early RSV LRTI, compared to 63% with an early rhinovirus illness, and 11% in those experiencing none of these severe infections. RSV LRTI cases had a lower response to bronchodilator tests and a smaller mean fractional concentration of exhaled nitric oxide (FENO) than those infected with human rhinovirus [71]. These observations support the role of early severe RSV infection in negatively modulating lung function throughout childhood. Other studies, however, suggest that severe RSV infections are instead a consequence of poor lung function. For example, Martinez et al. reported in 124 patients from Arizona decreased total respiratory conductance preceding severe LRTI and recurrent wheezing [72]. This finding was confirmed years later in 411 children in Copenhagen using neonatal spirometry [73].

## 5. Probe Studies Exploring Causality

Recent interventional studies, most of them in premature babies, suggest that severe RSV LRTI can contribute to the inception of recurrent wheezing. In fact, evidence today suggests a potential role for RSV prevention in decreasing its burden [8,9,74,75].

Various studies explored the preventive efficacy or effectiveness of palivizumab, an anti-RSV monoclonal antibody (mAb) administered to premature babies, against long term wheezing and asthma at ages one and six years [8,9,75,76].

The first evaluation of an intervention against RSV to protect the lungs from chronic injury was reported by Wenzel et al. in 2002. Investigators assessed a group of 13 high-risk children who had received immune globulin in infancy, in comparison to a control group of 26 high-risk children who received no early prophylaxis at seven to ten years of age. Drug recipients had a higher FEV1/FVC ratio, less atopy, and fewer asthma attacks [77].

A two-year observational study of 193 premature infants who received palivizumab and were not hospitalized for RSV detected a relative reduction in the proportion of children with recurrent wheezing and with physician-diagnosed recurrent wheezing, when compared to 231 children who did not receive the monoclonal antibody. Palivizumab prevented recurrent wheeze only in the subgroup of children with no history of allergic disease [78].

A follow up case-control study of 349 Japanese late-preterm infants who received palivizumab and 95 who did not observed a significant reduction in the incidence of recurrent wheezing at age three along with a trend towards reduction in IgE levels [9]. The reduction in physician-diagnosed recurrent wheezing persisted up to age six, but no differences were seen in the diagnosis of atopic asthma, defined as recurrent wheezing with an elevated IgE [79].

In the same year, a randomized controlled trial in the Netherlands explored differences in cumulative wheezing days and diagnosis of recurrent wheeze after palivizumab vs placebo administration in late premature infants. Investigators observed a relative reduction of 61% (CI 95% 56–65%) in total wheezing days in the palivizumab group [8]. Again, differences were observed in recurrent wheezing rates, but not in physician-diagnosed asthma or in spirometry tests at age six [80].

Interestingly, a similar study using a next generation palivizumab with higher affinity for the virus (i.e.,: motavizumab) in healthy Native Americans in Arizona prevented severe acute RSV LRTI, but did not affect the rates of medically attended wheezing in children aged 1–3 years [74]. Evidently, subjects and their pathways to wheezing are not always identical. Consequently, interventions may work well in certain groups and/or disease pathways, but not in others.

In recent years, a group of investigators elaborated guidelines for the evaluation of recurrent wheezing and asthma in ongoing vaccine and monoclonal antibody studies [81]. Yet, we must remain mindful that asthma is a set of heterogeneous diseases sharing a set of common symptoms. Therefore, it is possible that preventing severe RSV LRTI may contribute to decreasing one or a few of these asthma endotypes, but not others. Only a sophisticated discrimination of those endotypes under the “RSV LRTI and asthma umbrellas” will permit a thorough definition of the mechanistic associations between both syndromes.

## 6. Endotypes in RSV LRTI and Asthma

The diverse clinical presentations, the varying combination of signs and symptoms, and the heterogeneity in long-term outcomes suggest that RSV LRTI is not a single disease. For example, middle-class urban and suburban infants with loss-of-function single nucleotide polymorphisms in Asp299Gly and/or Thr399Ile (TLR4+/−) present exaggerated type 2 responses in the respiratory tract during RSV LRTI and experience severe disease with prolonged hospitalizations. Interestingly, premature infants from this population were not protected by the administration of palivizumab [55]. Additionally, children in the Navajo and Apache reservations that seem to be highly susceptible to acute infection with the virus, were solidly protected by monoclonal antibodies, but experienced no benefit in their rates of long term wheezing [74,82]. In line with these observations, Dumas et al. used an unsupervised model for probabilistic clustering (latent class analysis) to classify two cohorts of children admitted for bronchiolitis. When grouping children according to their clinical manifestations and viral etiology, they identified four previously unknown phenotypes of severe bronchiolitis [83]. Therefore, efficacy and subsequent effectiveness of preventive strategies against RSV may differ for certain RSV LRTI endotypes. A similar problem is posed by the diagnosis of asthma, a constellation of signs and symptoms modulated by different agents, environmental factors, and genetic predispositions. Characterization of specific RSV LRTI endotypes will probably help to overcome the need to fit this relationship in the dichotomous (yes or no) asthma definition at age six, and uncover new diseases within the two syndromes known as RSV bronchiolitis and asthma. This step forward will require data-driven statistical techniques in large cohorts of children, to define clusters based on clinical variables, biomarkers, responses to treatments, seasonality of exacerbations, lung function measurements, environmental exposures, and eventually, genetic studies.

## 7. Conclusions

RSV infection in infants and young children is associated with recurrent wheezing and impaired lung function. Symptoms improve throughout childhood. Abundant evidence suggests that RSV bronchiolitis is not a disease itself, but a composition of entities presenting clinically as a syndrome with partial differences in phenotypes and defined differences in disease mechanisms. Understanding the pathways to acute RSV disease will be important to better tie its inception and evolution to the acquisition of chronic wheezing disorders.
